# A GENERIC METHODOLOGY FOR EFFECTIVE CREATION OF LABORATORY-TEST-BASED FRAILTY INDICES

**DOI:** 10.1093/geroni/igz038.357

**Published:** 2019-11-08

**Authors:** Garrett M Stubbings, Arnold Mitnitski, Andrew Rutenberg

**Affiliations:** 1 Dalhousie University, Dartmouth, Nova Scotia, Canada; 2 Dalhousie University, Department of Medicine, Halifax, Nova Scotia, Canada; 3 Dalhousie University, Halifax, Nova Scotia, Canada

## Abstract

Laboratory test-based frailty indices (FI) are known to be highly predictive of adverse health outcomes and mortality. However, these FI depend on the proper classification of continuous valued health measurements into binary deficits (healthy or unhealthy). This classification is not standardized and is often done by using thresholds for medical intervention or maximally predictive values from statistical tests. This work proposes a simple and generic methodology for the creation of FI from laboratory values and measures its performance against existing methods. The methodology is as follows: a direction of risk is determined for each measurement, individuals are then assigned a score based on their relative standing in the population, binarization is then done using a global cut-point which binarizes all measures based on a given quantile. This method is shown to outperform FI created with medical risk thresholds for a range of global cut-points in both the NHANES and CSHA studies. Furthermore, our method is shown to be more robust to cohort effects than FI created using cut-points determined by maximum information measures, such as maximal separation of survival curves.

